# Evaluation of chronic lymphocytic leukemia by oligonucleotide-based microarray analysis uncovers novel aberrations not detected by FISH or cytogenetic analysis

**DOI:** 10.1186/1755-8166-4-25

**Published:** 2011-11-16

**Authors:** Kathryn A Kolquist, Roger A Schultz, Marilyn L Slovak, Lisa D McDaniel, Theresa C Brown, Raymond R Tubbs, James R Cook, Karl S Theil, Victoria Cawich, Caitlin Valentin, Sara Minier, Nicholas J Neill, Steve Byerly, S Annie Morton, Trilochan Sahoo, Blake C Ballif, Lisa G Shaffer

**Affiliations:** 1 Sacred Heart Medical Center, 101 West 8th Avenue, Spokane, WA, 99204, USA; 2 Signature Genomic Laboratories, PerkinElmer Inc., 2820 North Astor Street, Spokane, WA, 99207, USA; 3 CSI Laboratories, 11525 Park Woods Circle, Alpharetta, GA, 30005, USA; 4 Cleveland Clinic, Pathology & Laboratory Medicine Institute, 9500 Euclid Avenue, Cleveland, OH, 44195, USA; 5 Quest Diagnostics Nichols Institute, 14225 Newbrook Drive, Chantilly, VA, 20151, USA; 6 Quest Diagnostics Nichols Institute, 33608 Ortega Highway, San Juan Capistrano, CA, 92675, USA

**Keywords:** chronic lymphocytic leukemia, microarray, oligonucleotide, FISH, cytogenetics, chromosome aberration

## Abstract

**Background:**

Cytogenetic evaluation is a key component of the diagnosis and prognosis of chronic lymphocytic leukemia (CLL). We performed oligonucleotide-based comparative genomic hybridization microarray analysis on 34 samples with CLL and known abnormal karyotypes previously determined by cytogenetics and/or fluorescence *in situ *hybridization (FISH).

**Results:**

Using a custom designed microarray that targets >1800 genes involved in hematologic disease and other malignancies, we identified additional cryptic aberrations and novel findings in 59% of cases. These included gains and losses of genes associated with cell cycle regulation, apoptosis and susceptibility loci on 3p21.31, 5q35.2q35.3, 10q23.31q23.33, 11q22.3, and 22q11.23.

**Conclusions:**

Our results show that microarray analysis will detect known aberrations, including microscopic and cryptic alterations. In addition, novel genomic changes will be uncovered that may become important prognostic predictors or treatment targets for CLL in the future.

## Background

Chronic lymphocytic leukemia (CLL), the most common leukemia diagnosed in adults from Western countries, is characterized by a monoclonal population of mature activated B lymphocytes that usually express CD5+ and CD23+. However, the clinical features, disease course, and outcomes are highly variable. Most patients diagnosed with CLL can survive for many years, but in a subset of patients the course progresses more rapidly and is fatal despite aggressive treatment.

Currently, the diagnosis of CLL is made using histopathology; flow cytometry with a typical pattern of co-expression of CD5, CD23, CD20(dim), and surface Ig(dim); and chromosomal abnormalities detected by fluorescence *in situ *hybridization (FISH) probes and karyotyping. Conventional cytogenetics with karyotyping requires the use of an immunostimulatory CpG-oligodinucleotide DSP 30 plus IL-2 cocktail to enhance the yield of detectable chromosome aberrations in CLL cells. This cell culturing process is costly, time consuming, and requires the clinical indication of CLL at sample submission. Alternatively, FISH has been used to detect specific prognostic chromosome markers in CLL using a panel of five to six probes. However, locus-specific FISH does not reveal the complete cytogenetic picture [1]. Prognostic markers, determined primarily using FISH, include deletions of 13q, 17p, and 11q and trisomy 12. Most anomalies detected by cytogenetics in CLL are copy number gains and losses; translocations are rarely identified [2].

Previously, clinicians waited until patients diagnosed with CLL progressed to a specific stage to initiate therapy. Rai *et al*. [3,4] and Binet *et al*. [5] created staging systems that, until the past decade, were the hallmark for defining disease extent, prognosis, and initiation of treatment of CLL patients. Although these systems were standard of care, they did not predict the disease course for early-stage disease. In the past decade, our understanding of the pathophysiology of CLL has changed significantly with discoveries such as somatic mutations in the immunoglobulin heavy chain variable region (*IGHV) *genes, which are associated with a good prognosis [6,7], and lack of *IGHV *mutations and increased CD38 and ZAP-70 expression, which are associated with poor prognosis. Along with this has come a change in the way CLL has been approached therapeutically [8].

Microarray-based comparative genomic hybridization (aCGH) on neoplastic specimens has facilitated diagnosis and gene discovery with the ability to perform genome-wide investigations [7-18]. These array studies have shown concordance with the cytogenetic and FISH results. In addition, these studies demonstrated that appropriate microarray design can facilitate the detection of clinically relevant findings that would be missed using FISH panels. Although promising, most such microarray studies of CLL to date have been technically limited by the use of non-targeted BAC arrays [8,9,11], CGH-based oligonucleotide arrays that have either been non-targeted [10,19] or targeted to a relatively small subset of cancer genes or genomic regions (~15) associated with cancer [16,20], or SNP-based, whole genome (non-targeted) arrays [21].

In a novel approach as compared to these previous array studies, we developed a 135K-feature oligonucleotide-based microarray specifically targeted to more than 1800 cancer genes and regions and evaluated 34 patients diagnosed with CLL to compare the performance of this array to that of chromosome analysis and FISH. Our results further support microarray analysis as a diagnostic tool to detect cytogenetic abnormalities associated with CLL. In addition to the detection of known chromosomal rearrangements, we identified cryptic and novel DNA alterations using aCGH.

## Results

We tested 34 samples, which had been previously assessed by routine chromosome analysis and/or FISH, on a CGH-based microarray designed for detecting copy gains and losses associated with leukemia and lymphoma. Copy number alterations (CNAs) were identified in all 34 samples (Table 1). Ten of the cases had prior chromosome analysis, and all of these samples had an abnormal karyotype. By karyotype, the number of abnormalities detected ranged from 1 (trisomy 12) to 10 (complex with hypodiploidy) with an average number of 4 abnormalities detected per case. By comparison, among all 34 cases, the average number of abnormalities per case by microarray analysis was 4.4. We identified additional cryptic and/or novel aberrations by aCGH in 20 of 34 (59%) cases (Table 1).

**Table 1:** Thirty-four samples validated on an oligonucleotide array designed for detection of aberrations in leukemia and lymphoma*

Case	Karyotype	FISH		Concordant array results	Discordant array results	New findings by microarray
1	NA	LSI D13S319 96% loss, LSI 13q34 normal, LSI ATM normal, CEP 12 normal, LSI p53 normal		13q14.2q14.3(47,613,553-50,628,718) × 1	NA	22q11.23(22,674,846-22,723,991) × 0

2	NA	LSI D13S319 77% loss, LSI 13q34 normal, LSI ATM normal, CEP 12 normal, LSI p53 normal		13q14.2q14.3(47,123,245-50,443,082) × 1	NA	NA

3	NA	LSI D13S319 81% loss (34% mono, 47% bi), LSI 13q34 normal, LSI ATM normal, CEP 12 normal, LSI p53 normal		13q13.3q14.3(36,736,548-51,816,512) × 1, 13q14.3(49,315,855-50,237,971) × 0	NA	15q11.2(19,129,891-19,224,501) × 3, 18p11.32q23(123,388-76,100,854) × 3, 22q11.23(22,674,846-22,723,991) × 0

4	NA	LSI D13S319 42% loss, LSI 13q34 normal, LSI ATM normal, CEP 12 normal, LSI p53 normal		13q14.13q14.3(45,945,097-50,339,992) × 1	NA	NA

5	NA	LSI D13S319 normal, LSI 13q34 normal, LSI ATM normal, CEP 12 92% trisomy, LSI p53 normal		12p13.33q24.33(60,861-132,267,241) × 3	NA	NA

6	NA	LSI D13S319 97% loss, LSI 13q34 normal, LSI ATM normal, CEP 12 74% trisomy, LSI p53 normal		12p13.33q24.33(60,861-132,267,241) × 3, 13q14.3(49,074,574-50,628,718) × 1	NA	19p13.3q13.43(220,598-63,782,017) × 3

7	NA	LSI D13S319 91% loss, LSI 13q34 normal, LSI ATM 89% loss, CEP 12 normal, LSI p53 normal		11q13.4q24.3(72,459,008-130,030,128) × 1, 13q14.11q21.1(43,421,790-53,019,141) × 1	NA	2p25.3p11.2(44,198-89,912,901) × 3

8	NA	LSI D13S319 normal, LSI 13q34 normal, LSI ATM normal, CEP 12 63% trisomy, LSI p53 normal		12p13.33q24.33(60,861-132,267,241) × 3	NA	NA

9	NA	LSI D13S319 normal, LSI 13q34 normal, LSI ATM normal, CEP 12 63% trisomy, LSI p53 normal		12p13.33q24.33(60,861-132,267,241) × 3	NA	NA

10	NA	LSI D13S319 92% loss, LSI 13q34 12% loss, LSI ATM normal, CEP 12 normal, LSI p53 normal		13q14.3(49,407,720-50,523,594) × 0~1	Array did not detect 13q34 deletion seen in 12% of cells by FISH	12q24.12(110,684,027-110,768,579) × 3

11	NA		LSI D13S319 normal, LSI 13q34 normal, LSI ATM normal, CEP 12 normal, LSI p53 normal	NA	NA	1p31.3(64,811,810-68,404,781) × 3, 1q21.3q23.1(151,852,847-155,280,574) × 3, 2p16.1p15(59,245,962-62,672,016) × 3, 5q35.2q35.3(174,945,789-178,610,160) × 3, 11p15.4p15.3(7,907,684-11,413,676) × 3, 12p13.33p13.31(2,409,808-5,956,328) × 3, 16q24.1q24.2(82,968,178-86,062,471) × 3, 17q22q23.2(53,582,801-57,412,725) × 3, 18q21.32q22.1(57,089,022-60,760,895) × 3

12	NA		LSI D13S319 45% loss, LSI 13q34 normal, LSI ATM 47%, CEP 12 normal, LSI p53 normal	11q14.1q24.3(79,218,490-128,057,190) × 1, 13q14.11q21.33(43,525,071-71,879,067) × 1	NA	Yp11.31q12(2,706,656-57,735,230) × 0

13	NA		LSI D13S319 93% loss, LSI 13q34 normal, LSI ATM normal, CEP 12 normal, LSI p53 normal	13q14.3(49,457,877-50,339,992) × 0	NA	NA

14	NA		LSI D13S319 normal, LSI 13q34 normal, LSI ATM normal, CEP 12 71% trisomy, LSI p53 normal	12p13.33q24.33(60,861-132,267,241) × 3	NA	14q24.1q32.33 (68,329,913-105,393,508) × 1, 22q11.23(22,674,846-22,723,991) × 0

15	NA		LSI D13S319 89% loss, LSI 13q34 normal, LSI ATM 98% loss, CEP 12 normal, LSI p53 normal	11q14.3q23.2(88,551,231-114,026,260) × 1, 13q14.2q14.3(47,463,489-51,926,538) × 1	NA	2p16.1p14(56,499,065-66,570,230) × 3, 4p16.3p15.1(45,627-29,325,651) × 1, 5q33.2q35.3(152,262,081-180,619,169) × 3, 7q31.32q36.3(123,057,209-158,821,424) × 3

16	NA		LSI D13S319 40% loss, LSI 13q34 normal, LSI ATM normal, CEP 12 normal, LSI p53 normal	13q14.2q14.3(47,691,117-50,339,992) × 1	NA	1q32.1(203,529,401-204,498,513) × 1, 12p13.33q24.33(60,861-132,267,241) × 2~3

17	NA		LSI D13S319 92% loss, LSI 13q34 normal, LSI ATM 11% loss, CEP 12 90% trisomy, LSI p53 normal	12p13.33q24.33(60,861-132,267,241) × 3, 13q13.3q21.1(37,240,922-55,410,522) × 1	Array did not detect deletion of 11q22.3 seen in 11% of cells by FISH	5p15.32p15.31(5,388,368-6,879,401) × 1, 10q21.1q21.3(58,227,677-67,149,424) × 1, 10q23.31q23.33(90,215,922-95,066,500) × 1, 11q22.1q22.2(101,471,877-101,736,881) × 1, Yp11.32q12(1-57,735,230) × 0,

18	NA		LSI D13S319 70% loss, LSI 13q34 normal, LSI ATM 58% loss, CEP 12 normal, LSI p53 normal	11q14.1q25(78,733,283-131,062,293) × 1, 13q14.2q14.3(48,739,670-50,554,228) × 1	NA	NA

19	NA		LSI D13S319 96% loss, LSI 13q34 normal, LSI ATM 98% loss, CEP 12 normal, LSI p53 normal	11q14.3q23.3 (91,814,326-116,080,874) × 1, 13q14.2q14.3(48,774,702-50,765,417) × 1	NA	2p25.3p14(44,198-66,539,084) × 3, 2p14p11.2(66,729,955-88,771,193) × 2~3, 4q32.3q35.2(166,094,098-191,152,793) × 1, 6q16.3q27(102,816,244-170,736,131) × 1~2, 7p22.1p11.2(6,480,544-55,435,373) × 2~3, 8q23.1q24.3(107,914,570-146,263,042) × 3, 11p15.1p14.3(21,533,469-22,367,835) × 1, 11p14.3(22,398,459-24,320,961) × 3, 13q13.3(37,240,922-38,696,855) × 1, 13q14.11q14.12(43,995,777-5,551,120) × 3, 13q14.3q21.1(50,795,724-52,401,010) × 3, 13q21.1q21.2(55,083,523-58,084,123) × 3, 13q21.2(59,114,933-59,262,005) × 3, 13q21.31q21.32(63,478,713-65,130,349) × 3, 13q21.33q34(67,650,203-114,103,644) × 3, 19p13.3(220,598-546,817) × 1, 21q22.3(42,101,144-46,915,771) × 2~3

20	NA		LSI D13S319 normal, LSI 13q34 normal, LSI ATM normal, CEP 12 66% trisomy, LSI p53 62% loss	12p13.33q24.33(60,861-132,267,241) × 3, 17p13.3p11.1 (49,128-22,116,415) × 1	NA	22q11.23(22,674,846-22,731,268) × 0~1, Yp11.2q11.23(8,292,949-26,636,748) × 0

21	NA		LSI D13S319 92% loss, LSI 13q34 normal, LSI ATM normal, CEP 12 normal, LSI p53 normal	13q14.2q14.3(46,444,224-50,554,228) × 1	NA	NA

22	NA		LSI D13S319 normal, LSI 13q34 normal, LSI ATM normal, CEP 12 35% trisomy, LSI p53 normal	12p13.33q24.33 (60,861-132,267,241) × 3	NA	NA

23	NA		LSI D13S319 29% loss, LSI 13q34 normal, LSI ATM normal, CEP 12 normal, LSI p53 normal	13q14.3(49,604,393-49,730,034) × 1	NA	NA

24	NA		LSI D13S319 88% loss, LSI 13q34 normal, LSI ATM normal, CEP 12 normal, LSI p53 normal	13q14.2q14.3(46,444,224-50,554,228) × 1	NA	NA

25	44~46,X,X,add(1)(p36.1),i(6)(p10),del(8)(p21p23),add(12)(p11.2),**-**13, del(14)(q12q32),-15, add(17)(p11.2),+2-6mar[cp11]/46,XX[9]		LSI D13S319 34% loss, LSI p53 49% loss	13q14.2q14.3(47,524,866-50,523,594) × 1, 17p13.3p13.1(49,128-8,581,862) × 1	Array did not detect del(14)(q12q32) seen on karyotype; -15 shows complexity on array	3p21.31(47,218,579-49,437,299) × 1, 5q35.1q35.3(168,342,673-180,619,169) × 3, 6p21.33p12.1(31,649,559-55,535,574) × 1, 7p22.3p15.3(130,978-21,156,763) × 3, 8p23.3p12(177,781-34,695,588) × 1, 8q24.13q24.3(126,709,259-145,344,434) × 3, 15q11.2q13.3(20,372,901-31,359,613) × 3, 15q13.3q15.1(31,388,923-39,994,112) × 1, 15q15.1q21.1(40,049,653-43,331,117) × 3, 15q21.1q22.2(43,385,070-58,614,700) × 1, 15q22.2q26.3(58,646,551-100,217,531) × 3

26	47,XY,+12[cp7]/46,XY[13]		LSI D13S319 normal, LSI 13q34 normal, LSI ATM normal, CEP 12 77.5% trisomy, LSI p53 normal	12p13.33q24.33(60,861-132,267,241) × 3	NA	7q34(141,693,456-141,719,136) × 1, 14q22.2(53,498,118-53,858,816) × 3

27	46,XY,del(11)(q13q23)[5]/47,idem,+12[8]/46,XY[7]		LSI D13S319 67% loss, LSI ATM 93% loss, CEP 12 15% trisomy	11q13.5q23.3(76,383,882-117,091,784) × 1, 12p13.33q24.33(1-132,349,534) × 3, 13q14.2q14.3(47,172,707-50,586,402) × 1~2	NA	NA

28	47,XY,+12[6]/46,XY[14]		LSI D13S319 normal, LSI 13q34 normal, LSI ATM normal, CEP 12 61% trisomy, LSI p53 normal	12p13.33q24.33(60,861-132,267,241) × 3	NA	NA

29	45,XY,del(13)(q21q34),-17[3]/46,XY,del(17)(p11.2)[3]/46,XY[14]		LSI D13S319 normal, LSI ATM normal, CEP 12 normal, LSI p53 45% loss	17p13.3p11.2(49,128-21,376,245) × 1	Array and FISH did not detect del(13)(q21q34) seen in 3/20 cells by karyotype	NA

30	47,XY,**+**12[4]/47,idem,del(11)(q13q23)[4]/46,XY[8]		LSI D13S319 normal, LSI 13q34 normal, LSI ATM 10.5% loss, CEP 12 85% trisomy, LSI p53 normal	12p13.33q24.33(60,861-132,267,241) × 3	Array did not detect 11q22.3 deletion seen in 10.5% of cells by FISH and in 4/16 cells by karyotype	13q12.2(27,492,830-27,493,700) × 3, 22q11.23(22,674,846-22,731,268) × 0

31	44~46,XY,del(6)(q15q23)[6]/42~44, idem,-3,-4,add(4)(p14),-8,add(10)(q22),del(13)(q12q22),add(17)(p11.2)[cp14]		NA	3p26.3p25.2(88,832-11,601,487) × 1, 3p24.3p21.31(21,821,826-45,382,789) × 1, 3p21.2p14.2(51,631,148-59,828,728) × 1, 3p11.2q21.3(88,616,646-129,821,181) × 1, 3q21.3q25.1(130,955,610-152,751,429) × 1, 4p16.3p14(45,627-39,064,794) × 1, 4p14p12(40,689,654-48,288,082) × 1, 6q14.1q24.3(83,060,798-147,519,394) × 1, 8p23.3p21.3(1-21,077,797) × 1, 8p21.1q12.1(27,859,739-56,426,895) × 1, 8q12.1q13.1(58,060,709-66,596,062) × 1, 8q21.13q22.1(81,697,427-96,822,423) × 1, 8q22.3q24.13(103,345,568-125,046,236) × 1, 8q24.21q24.3(130,132,793-141,102,164) × 1, 13q14.12q34(45,300,002-114,103,644) × 1	NA	10q24.1q25.2(99,015,708-114,057,326) × 1, 11q22.1q23.3(101,560,685-116,463,713) × 1, 16p13.3p13.2(35,819-8,125,327) × 1, 16p13.13p13.12(12,434,601-14,674,345) × 1, 16q21(57,250,542-61,322,202) × 1, 17p13.3p11.2(49,128-21,376,245) × 1, 18q12.3(38,266,239-38,764,068) × 1, 20p13p12.2(517,864-11,841,120) × 1; Array clarified a deletion on 13q to include *LAMP1*

32	45~46,XX,del(17)(p11.2p13),-20, +mar[cp16]/45~46,idem,add(3)(q25)[3]/46,XY[3]		LSI D13S319 72% loss, LSI 13q34 normal, LSI ATM normal, CEP 12 normal, LSI p53 79.5% loss	13q14.3(49,378,768-50,262,893) × 1, 17p13.3p11.2(49,128-21,247,183) × 1, 20p13p12.1(1,981,763-15,398,390) × 1, 20p11.21(23,271,365-25,666,747) × 1, 20q11.21q11.22(30,436,259-32,563,890) × 1	NA	3p21.31(46,911,912-49,973,212) × 1, 17q21.1q21.31(35,551,063-37,865,072) × 1

33	45,XY,-4, add(17)(p13)[1]/44,idem,add(13)(p12),add(14)(q32),**-**15, del(20)(q11.2q13.3)[16]/46,XY[3]		NA	4p16.3p14(45,627-38,504,396) × 1, 4p13q22.3(41,631,408-96,524,997) × 1, 15q11.2q15.1(19,129,891-38,918,282) × 1, 15q21.2q22.2(49,131,112-57,212,181) × 1	del(20)(q11.2q13.3) not detected by aCGH	11q22.3q23.2(106,821,962-113,825,965) × 1, 13q12.11(19,508,097-20,625,750) × 1, 17p13.3(49,128-2,779,693) × 1, 17p13.1p11.2(9,888,292-18,868,118) × 1, 17p11.2(19,082,873-20,794,597) × 1, 22q11.23(22,674,846-22,723,991) × 0

34	45,X,-Y[7]/46,XY,add(8)(p11.2),add(11)(q13)[4]/46,XY[9]		LSI D13S319 62.5% loss, LSI 13q34 normal, LSI ATM 31% loss, CEP 12 normal, LSI p53 normal	11q13.4q25(72,590,406-134,425,038) × 1, 13q14.2q14.3(47,588,669-50,414,293) × 1	-Y not detected by aCGH	7p22.3p12.2(130,978-49,815,456) × 3, 8p23.3p12(1-33,376,370) × 1, 22q12.2q13.33(30,406,286-49,519,766) × 3

aCGH, array-based comparative genomic hybridization; NA, not applicable.

*Rearrangements at the immunoglobulin loci were excluded except for α T-cell receptor loci (13q14.2) and β T-cell receptor loci (7q34) since they can recombine in malignant B cells [19].

## Discussion

### Common aberrations identified by microarray analysis

Our results confirm prior studies that show the most common abnormalities found in CLL and identifiable by arrays are deletion of 13q14.3, trisomy 12, deletion of 11q22.3 and deletion of 17p13.1.

**Figure 1:**
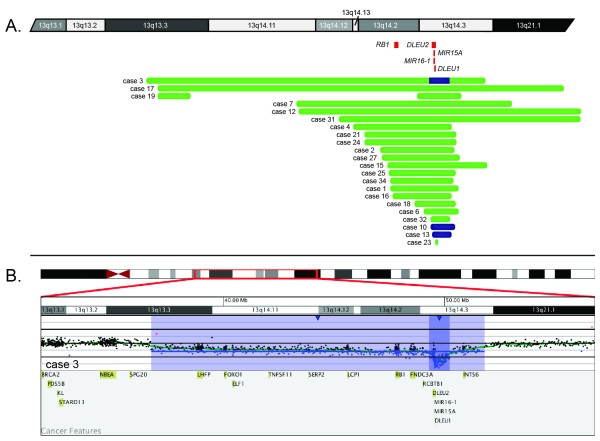
**Microdeletions of 13q14.3 detected by microarray analysis**. (A) Green bars represent deletion sizes for each case (based on UCSC 2006 hg18 assembly). Cases 3, 10 and 13 had biallelic deletions, represented by navy blue bars. Red boxes represent genes of interest in the interval. (B) Microarray results for case 3. Microarray analysis showed biallelic deletion of *MIR15A/MIR16-1, DLEU2*, and *DLEU1 *(shaded in dark blue) and monoallelic deletion of *RB1 *(shaded in light blue). Probes are ordered on the *x*-axis according to physical mapping positions, with the most proximal 13q probes on the left and the most distal 13q probes on the right. Values along the *y*-axis represent log_2 _ratios of patient:control signal intensities. Results are visualized using Oncoglyphix (Signature Genomics).

The 13q14 region has been identified as a recombination hot spot [9] and includes the *RB1, DLEU1*, and *DLEU2 *genes and microRNAs *MIR16-1 *and *MIR15A*. Deletion of the 13q14.3 region distal to *RB1 *is the most common chromosomal abnormality found in CLL [10], and the *DLEU2/MIR15A/MIR16-1 *locus has been shown to play a role in controlling the expansion of mature B cells by down-regulating the genes that control entry into the cell cycle [11]. Twenty-two cases had known deletions of 13q by karyotyping and/or FISH. Figure 1A shows the 22 cases in which 13q deletions were detected by microarray analysis. Nineteen samples had a monoallelic deletion at 13q14.3 ranging in size from 0.12 to 68.8 Mb, and three of the samples had a biallelic deletion of 13q14.3 ranging in size from 0.88 to 1.12 Mb. Of the 19 cases with monoallelic deletions, 15 had deletion of *RB1, MIR15A, MIR16-1, DLEU2, DLEU1*; two had deletion of *DLEU2, DLEU1, MIR15A *and *MIR16-1 *without deletion of *RB1*; and one had deletion of *DLEU1, MIR15A *and *MIR16-1 *without deletion of *RB1 *or *DLEU2*. Case 23 had a 126-kb deletion that did not include *RB1, MIR15A, MIR16-1, DLEU2 *or *DLEU1*, although the deletion was seen in 29% of nuclei by FISH. Of the three biallelic deletions, two retained *RB1 *while *MIR15A, MIR16-1, DLEU2 *and *DLEU1 *were deleted, and one had biallelic deletion of *MIR15A, MIR16-1, DLEU2*, and *DLEU1 *and monoallelic deletion of *RB1 *(Figure 1B). Case 19 showed a complex and cryptic pattern with deletions of 13q13.3 and 13q14.2q14.3 and duplications of 13q14.11q14.12, 13q14.3q21.1, 13q21.1q21.2, 13q21.2, 13q21.31q21.32 and 13q21.33q34. Thus, our data and that of others[22] confirm that aCGH can delineate the sizes and complexities of 13q deletions better than conventional cytogenetic and FISH analyses. However, based on FISH analysis [23], there appears to be no difference in overall survival between patients with monoalleleic and bialleleic deletions. The precision gained in delineating breakpoints and the genomic content in regions of deletions using microarrays has the potential to uncover additional genomic variation in these patients that might be a better predictor of overall survival than what can be understood based on FISH. For example, it has been suggested that genomic variation may offer insight into the potential aggressive behaviors for the disease [12]. Larger deletions that include the *RB1 *locus have been proposed to be associated with greater genomic complexity and a more aggressive course. However, in the current study, several cases (e.g., cases 19 and 32) had 13q14 deletions that did not encompass *RB1 *and still presented with complex findings, and other cases had deletions that encompassed *RB1 *but lacked significant complexity. Further studies are warranted to determine the prognostic value of sizing 13q deletions.

All trisomy 12-positive cases were detected by microarray analysis. The percentage of abnormal cells detected ranged from 15% to 92% (as determined by FISH). Trisomy 12 was identified by microarray analysis in one additional case in which FISH analysis was normal (case 16); follow-up studies on this case were not possible.

Nine cases had known deletions of 11q22.3/*ATM *as determined by FISH and/or karyotype. Eight of these were identified by aCGH. The deletions ranged in size from 0.27 Mb to 61.8 Mb. In case 17 with an 11q22.3 deletion not detected by aCGH, the deletion was present in 11% of the interphase cells scored by FISH. Deletions of 11q that include *ATM *were found by aCGH in two cases that were missed by karyotype: case 31 had a 14.9-Mb deletion at 11q22.1q23.3, and case 33 had a 7-Mb deletion at 11q22.3q23.2. FISH was not performed in either case.

These cases with commonly found abnormalities illustrate the ability and limitations of aCGH to detect a relatively low number of cells with the abnormal clone (~15%) and to detect aberrations missed by conventional chromosome analysis and FISH. The high-density coverage afforded by the array design used in the current study likely contributes to both improved sensitivity in detecting known lower-level mosaic alterations and an improved ability to recognize new alterations. However, lower-level limits of resolution do persist for aCGH due to the nature of the technology. Thus, even with the improvements described herein, this assay should be used only for new diagnoses or relapse and not for monitoring for minimal residual disease.

### Microarray analysis can clarify the karyotypes

Karyotype complexity can be delineated by aCGH. For example, case 25 had a reported karyotype of monosomy 15 with two to six markers. Microarray analysis identified a series of gains and losses that included 15q11.2q13.3 × 3, 15q13.3q15.1 × 1, 15q15.1q21.1 × 3, 15q21.1q22.2 × 1, and 15q22.2q26.3 × 3. This result may represent the markers seen by karyotyping, although FISH was not performed to confirm these findings. For some cases in which karyotyping was not performed, we found highly complex genomic changes (e.g., cases 11 and 19).

In several cases, karyotyping showed abnormalities that should have been detected by microarray analysis but were not (Table 1). For example in case 29, karyotyping showed deletion of 13q that was not detected by array or locus-specific FISH, and in case 33 karyotyping showed a deletion at 20q11.2q13.3 in 16 of 20 cells that was not identified by microarray analysis. These results may indicate that the karyotype was not interpreted correctly and that no deletions are present at these loci.

Finally, aCGH clarified the deletion/rearrangement of 17p13.1 identified by prior karyotyping in two cases (cases 31 and 33). In both cases, microarray analysis identified a deletion of 17p. In case 31, the deletion encompasses *TP53 *that by karyotyping had been interpreted as an add(17p). Case 33 also had an add(17p) by karyotype analysis. Array analysis showed a deletion of 17p, but the deletion does not include *TP53*. Deletion of 17p is considered an independent prognostic factor with resistance to treatment, shorter treatment-free interval, and shorter overall survival [17]. Thus, aCGH can clarify the chromosome results, and in some cases, the identification of a deletion involving *TP53 *would change the prognosis for the patient and may be used to alter treatment or patient management. Additional cases in which the array results changed the prognosis are discussed in the following section.

### New prognostic information obtained by microarray analysis

In addition to common aberrations, we identified clinically significant or potentially significant gains or losses that were not known prior to submitting the sample for array analysis in the majority of cases (20/34), including trisomy 18 and 19 and deletions of 6q, which are highlighted here.

Trisomy 18 and trisomy 19, each seen in separate cases in our study, are uncommon in CLL. Trisomy 18 generally presents as the sole abnormality or with a karyotype that includes trisomy 12 or trisomy 19 [18]. Trisomy 19 in addition to trisomy 12 has been associated with *IGHV *gene mutation [13]. In that study of 705 cases of CLL, trisomy 19 was seen in 11 (1.6%) cases, all of which also had trisomy 12; nine had mutated *IGHV *genes. Those cases that did not have trisomy 19 but had trisomy 12 primarily had unmutated *IGHV *genes [13]. In our study, trisomy 12 was seen with trisomy 19 in case 6, although the *IGHV *mutational status is not known. In case 3 with trisomy 18, neither trisomy 12 nor 19 was detected.

Large (~64 Mb) deletions of 6q were detected in two cases, one of which, case 19, was not known prior to array analysis presumably because chromosome analysis was not performed. Cases of CLL with deletions of 6q are characterized by atypical lymphocyte morphology, CD38 positivity, and intermediate incidence of *IgVH *somatic hypermutation [14]. Cases of CLL with deletion of 6q (specifically at 6q21) are seen in less than 5% of CLL cases, have been shown to require a more demanding treatment regimen, and have been suggested to comprise an intermediate-risk group [14,15]. Deletion of 6q with or without other abnormalities may also be predictive of shorter survival [15].

In four cases (cases 7, 11, 15 and 19) for which only limited FISH was performed prior to aCGH analysis, new information was revealed and showed gains of 2p, which encompassed 2p16.1p15, ranging in size from 3.4 Mb to 89.8 Mb that included the *REL *and *BCL11A *genes. Gains in the 2p16.1p15 region have been associated with a poor prognosis and have been seen more frequently in cases that have deletion 17p- [16], although we did not see this association. The ability to perform microarray analysis on residual or archived material provides an opportunity to analyze the cancer genome in an unbiased and comprehensive approach.

### Novel aberrations identified by microarray analysis

We found 10 novel changes by microarray analysis that were not identified by karyotype or FISH and that may have clinical significance. For example in case 17, microarray detected additional losses, including a 265-kb loss on 11q that included *BIRC3*. This gene is part of the inhibitor of apoptosis (IAP) family, which plays a role in apoptosis and the inflammatory process [24] and, when fused by translocation to *MALT1*, is associated with MALT-type lymphoma [25]. Interestingly, we have identified a novel translocation of *BIRC3 *to *SETBP1 *in a separate case of CLL (unpublished observation). Furthermore, an NF-ĸB inhibitor has recently been shown to achieve apoptosis induction with potential therapeutic value for CLL in cases with reduced expression of *BIRC3 *[26]. This suggests that *BIRC3 *status may be an important factor in determining appropriate therapy and prognosis. However, further investigations are warranted. A recent publication reported atypical deletions of 11q in patients with CLL [27]. However, their minimal deletion region, as established by BAC array analysis, did not include *BIRC3*, as found in case 17 in our study.

**Figure 2:**
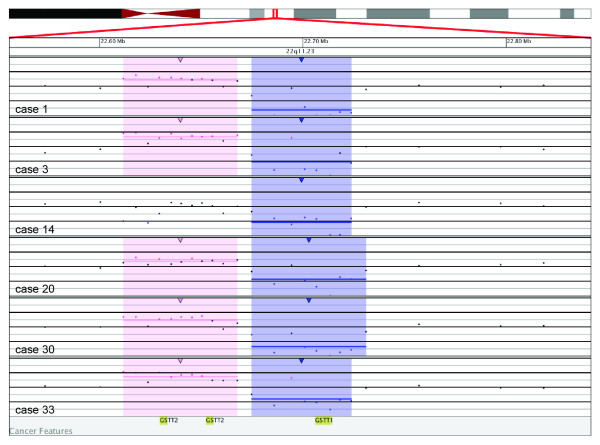
**Microarray results for six cases (cases 1, 3, 14, 20, 30 and 33) with a biallelic deletion at 22q11.23 of 49 to 56 kb that includes *GSTT1***. Probes are ordered on the *x*-axis according to physical mapping positions, with the most proximal 22q probes on the left and the most distal 22q probes on the right. Values along the *y*-axis represent log_2 _ratios of patient:control signal intensities. Results are visualized using Oncoglyphix (Signature Genomics).

In six cases (cases 1, 3, 14, 20, 30 and 33), a biallelic deletion at 22q11.23 of 49 to 56 kb that includes *GSTT1 *was found by array only (Figure 2). *GSTT1*, along with *GSTM1 *and *GSTP1*, is part of the glutathione S-transferase family, which encodes for enzymes that catalyze the conjugation of reduced glutathione to a variety of electrophilic and hydrophobic compounds. The enzyme activity of *GSTT1 *towards methyl chloride in erythrocytes can be measured and placed into three groups: nonconjugators, low conjugators, and high conjugators. Nonconjugators are assumed to have the *GSTT1*-null genotype and have been noted to have increased genotoxic affects such as sister chromatid exchanges after exposure to toxic agents such as methyl bromide [28]. Individuals with the *GSTT1*-null genotype have been shown to be at a increased risk for developing MDS [22], and polymorphisms in *GSTM1 *and *GSTP *are associated with a higher risk of developing CLL [29]. Based on BAC-array analysis, Gunn and coworkers [30] identified deletions of 22q11.22 involving the genes *GGTLC2 *and *PRAME*. This region is proximal to and appears non-overlapping with the novel deletions reported here of *GSTT1 *in 22q11.23.

**Figure 3:**
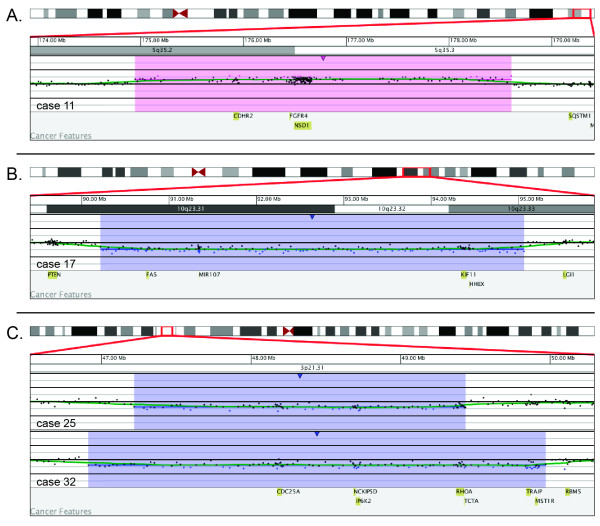
**Novel aberrations by microarray**. (A) Microarray results for case 11 showing a 3.6-Mb gain (shaded in pink) encompassing 5q35.2q35.3 that includes *CDHR2*, a tumor suppressor candidate. Probes are ordered on the *x*-axis according to physical mapping positions, with the most proximal 5q probes on the left and the most distal 5q probes on the right. (B) Microarray results for case 17 showing a 4.8-Mb deletion (shaded in blue) at 10q23.31q23.33 that includes *MIR107 *and *FAS*. Probes are ordered on the *x*-axis according to physical mapping positions, with the most proximal 10q probes on the left and the most distal 10q probes on the right. (C) Microarray results for cases 25 and 32 with deletions (shaded in blue) of 3p21.31 that include *CDC25A*. Case 25 has a 2.2-Mb deletion, and case 32 has a 3.1-Mb deletion. *CDC25A *is required for progression from G1 to S phase in the cell cycle. Probes are ordered on the *x*-axis according to physical mapping positions, with the most distal 3p probes on the left and the most proximal 3p probes on the right. For A–C, values along the *y*-axis represent log_2 _ratios of patient:control signal intensities. Results are visualized using Oncoglyphix (Signature Genomics).

Additional, novel aberrations included case 11 that had a 3.6-Mb gain that encompassed 5q35.2q35.3 and included *CDHR2 *(Figure 3A), a tumor suppressor candidate [31]. In case 17, a 4.8-Mb deletion at 10q23.31q23.33 was identified that includes *MIR107 *and *FAS *(Figure 3B). *MIR107 *plays a role in inhibiting differentiation in granulocytic, monocytic, and B-lymphoid lines [32], whereas *FAS *is involved with apoptosis, and mutations in *FAS *are known to cause autoimmune lymphoproliferative syndrome [33]. Cases 25 and 32 had a deletion at 3p21.31 of 2.2 Mb and 3.06 Mb, respectively, that included *CDC25A *(Figure 3C), which is required for progression from G1 to S phase in the cell cycle [34]. Both cases 25 and 32 exhibit deletions of *MIR15A/MIR16-1 *and *TP53*, but not *ATM*. This may prove to be related to the acquisition and/or significance of the *CDC25A *deletion. Identification of additional cases with deletions of these novel genes may assist in understanding their potential roles in CLL.

## Conclusions

We have used a novel approach of targeting over 1800 cancer feature genes while also providing whole genome coverage to identify novel changes and delineate breakpoints of alterations. Using this approach, we have shown that such an array design in CLL will identify cryptic and novel alterations, clarify the karyotype results and refine breakpoints, which may lead to better prognostic precision in CLL, and may influence treatment or patient management. This approach is likely superior to using small, targeted arrays that may miss important novel changes or high-density, whole-genome arrays that have arbitrary coverage and may yield findings that are difficult to interpret in the context of the patient's disease. Arrays also may be useful for cases in which the chromosome analysis and FISH results are discordant with each other, with the pathology, or with disease course. Because the array uses DNA extracted directly from the specimen, aCGH may be useful for cases of tissue culture failure. Finally, microarrays may be helpful when cytogenetics is negative or ambiguous. Because CLL rarely involves balanced translocations, which are not detectable by aCGH, this technology may be particularly useful for these patients, especially in understanding the cancer genomes for the 10–20% of cases representing young patients [35], who exhibit a significantly reduced life expectancy relative to healthy controls once symptomatic with this disease [36].

## Materials and methods

### DNA extraction

DNA was extracted from 34 samples collected from patients with either newly diagnosed CLL or recurrent disease. All samples were karyotypically abnormal by conventional cytogenetics, FISH, or both. The samples consisted of 22 peripheral bloods, 11 bone marrow aspirates, and one lymph node. The specimens were de-identified for demographic details but retained data regarding prior chromosome and FISH analyses. The protocol of testing of de-identified, discarded specimens was approved by the Institutional Review Board (IRB) Spokane, and IRB approval was obtained by the source laboratories where required by local regulations.

Genomic DNA was extracted from unenriched blood and bone marrow specimens using the Gentra Puregene Blood kit (Qiagen, Germantown, MD) according to the manufacturer's instructions. Two million cells or 150 μl (if cell counts were unavailable) of blood or bone marrow were used as starting material. Additional cell lysis solution (Gentra Puregene Blood kit) was added to samples with high viscosity to ensure complete cell lysis. Samples were stabilized in cell lysis solution within 24–48 hours when possible to ensure high-quality DNA for use on the microarray.

DNA quality was assessed by measuring DNA concentration, 260/280 and 260/230 readings on a Nanodrop 2000 Spectrophotometer (Thermo Scientific, Waltham, MA). The DNA was also run on a 1% agarose gel with ethidium bromide to determine if degradation was present. To be included in the study, samples had to have minimal degradation with 260/280 values near 1.8 and 260/230 readings greater than 1.35.

### Oligonucleotide microarray labeling, hybridization, and analysis

Oligonucleotide-based microarray analysis was performed using a 135K-feature whole-genome microarray (Signature OncoChip™, designed by Signature Genomic Laboratories, Spokane, WA; manufactured by Roche NimbleGen, Madison, WI). This microarray targets 1893 cancer features, including genes with known roles in hematologic malignancies or solid tumors in which deletions or mutations had been previously reported; genes with suspected roles in cancer based on prior expression studies without specific evidence of genomic copy changes; genes with previously speculated roles based solely upon association with a biological pathway or gene family; and genes involved in protein and miRNA coding. The microarray has an average oligonucleotide coverage of one oligo per 0.2–7 kb for targeted cancer features with additional genomic backbone coverage of approximately one oligo per 35 kb. Purified genomic DNA from the diagnostic specimens was labeled with Cyanine dye Cy5, and DNA from a chromosomally normal control was labeled with Cyanine dye Cy3, using a Roche NimbleGen Dual-Color DNA Labeling Kit according to the manufacturer's instructions. Array hybridization and washing were performed as specified by the manufacturer (Roche NimbleGen). Arrays were scanned at 5 microns using an MS 200 Microarray Scanner (Roche NimbleGen) and analyzed using MS 200 1.0 Scanning Software (Roche NimbleGen), NG Packager 1.0 (Signature Genomics) and NimbleScan 2.6 (Roche NimbleGen). Results were then displayed using custom oligonucleotide aCGH analysis software (Oncoglyphix™, Signature Genomics).

## Competing interests

RAS, LDM, VC, CV, SM, NJN, SB, SAM, BCB, and LGS are employees of Signature Genomic Laboratories, PerkinElmer, Inc. TS and MLS are employees of Quest Diagnostics. TCB is an employee of CSI Laboratories. The remaining authors have no conflict of interest to report.

## Authors' contributions

KAK, LGS, and AT wrote the manuscript; KAK, RAS, MLS, BCB, and LGS analyzed and interpreted the data; LGS, BCB, MLS, RAS, and TS designed the research; TCB, RRT, JRC, and KST contributed study samples; VC, CV, SM, NJN, SB, and SAM performed the research; LDM contributed to the discussion of research and data; and all authors critically reviewed and gave final approval of the manuscript.
